# Relationship between delivery with anesthesia and postpartum depression: The Japan Environment and Children’s Study (JECS)

**DOI:** 10.1186/s12884-021-03996-y

**Published:** 2021-07-23

**Authors:** Nobuhiro Suzumori, Takeshi Ebara, Hazuki Tamada, Taro Matsuki, Hirotaka Sato, Sayaka Kato, Shinji Saitoh, Michihiro Kamijima, Mayumi Sugiura-Ogasawara, the Japan Environment, Children’s Study Group, Shin Yamazaki, Yukihiro Ohya, Reiko Kishi, Nobuo Yaegashi, Koichi Hashimoto, Chisato Mori, Shuichi Ito, Zentaro Yamagata, Hidekuni Inadera, Takeo Nakayama, Hiroyasu Iso, Masayuki Shima, Youichi Kurozawa, Narufumi Suganuma, Koichi Kusuhara, Takahiko Katoh

**Affiliations:** 1 Department of Obstetrics and Gynecology, Nagoya City University Graduate School of Medical Sciences, 1 Kawasumi, Mizho-cho, Mizuho-ku, Nagoya, 467-8601 Japan; 2 Occupational and Environmental Health, Nagoya City University Graduate School of Medical Sciences, 1 Kawasumi, Mizho-cho, Mizuho-ku, Nagoya, 467-8601 Japan; 3 Pediatrics and Neonatology, Nagoya City University Graduate School of Medical Sciences, 1 Kawasumi, Mizho-cho, Mizuho-ku, Nagoya, 467-8601 Japan

**Keywords:** Anesthesia, Depression, Delivery, EPDS, Postpartum

## Abstract

**Background:**

Postpartum depression is one of the most commonly experienced psychological disorders for women after childbirth, usually occurring within one year. This study aimed to clarify whether women with delivery with anesthesia, including epidural analgesia, spinal-epidural analgesia, and paracervical block, had a decreased risk of postpartum depression after giving birth in Japan.

**Methods:**

The Japan Environment and Children’s Study (JECS) was a prospective cohort study that enrolled registered fetal records (n = 104,065) in 15 regions nationwide in Japan. Binomial logistic regression analyses were performed to calculate the adjusted odd ratios (aORs) for the association between mode of delivery with or without anesthesia and postpartum depression at one-, six- and twelve-months after childbirth.

**Results:**

At six months after childbirth, vaginal delivery with anesthesia was associated with a higher risk of postpartum depression (aOR: 1.233, 95% confidence interval: 1.079–1.409), compared with vaginal delivery without analgesia. Nevertheless, the risk dropped off one year after delivery. Among the pregnant women who requested delivery with anesthesia, 5.1% had a positive Kessler-6 scale (K6) score for depression before the first trimester (*p* < 0.001), which was significantly higher than the proportions in the vaginal delivery without analgesia (3.5%).

**Conclusions:**

Our data suggested that the risk of postpartum depression at six months after childbirth tended to be increased after vaginal delivery with anesthesia, compared with vaginal delivery without analgesia. Requests for delivery with anesthesia continue to be relatively uncommon in Japan, and women who make such requests might be more likely to experience postpartum depressive symptoms because of underlying maternal environmental statuses.

## Background

Epidural anesthesia during delivery is the most common and widely accepted method of pain relief during labor [1–3]. Although the proportion of pregnant women requesting pain control with anesthesia varies internationally between 20%-70% [2, 3], the proportion in Japan continues to be relatively low. Nevertheless, the use of anesthesia has recently been growing in popularity, and the proportion of women who use anesthesia during labor reportedly increased from 4.6% in 2014 to 6.1% in 2016 [4]. Since a common maternal myth in Japan is that labor pains are conducive to forming a strong maternal instinct [5], we assumed that this belief might be one of the reasons why delivery with anesthesia is uncommon in Japan, compared with other countries.

Giving birth in a more relaxed state through the use of anesthesia during delivery can be expected to confer benefits to both mother and baby. Even in healthy mothers, suppressing hyperventilation arising from pain and suppressing the deterioration in blood flow to the placenta as a result of the release of stress hormones are possible merits of epidural delivery. Analgesia and the accompanying reduction in childbirth stress might be particularly beneficial to mothers with chronic diseases, such as cardiovascular disease.

On the other hand, a recent report suggested increased risks in obstetric and neonatal outcomes among pregnant women with combined spinal-epidural analgesia during labor, compared with women without anesthesia; these risks included a prolonged duration of labor, instrumental delivery, lower Apgar scores, and an umbilical arterial blood gas pH of less than 7.10 [4]. In contrast, several lines of evidence suggest that epidural analgesia is associated with a decreased risk for postpartum depression [6–8], although the sample sizes of some prospective cohorts were limited.

Severe labor pains are risk factor for postpartum depression in pregnant women, and early depression is associated with an increased risk of long-term depression [9]. Postpartum depression affects women who have given birth and is a common disorder for new mothers. Almost 10% to 15% of mothers may suffer from postpartum depression within the first year after delivery [9]. Multiple factors may be involved in postpartum depression, and the causes have been difficult to understand.

The present study aimed to clarify whether vaginal delivery with or without analgesia, decreased the risk of postpartum depression after childbirth in Japan.

## Methods

### Study population

The design of the Japan Environment and Children’s Study (JECS) has been described previously in detail [10–12]. The direct web link to the JECS is https://www.env.go.jp/chemi/ceh/en/index.html This study followed the STROBE (Strengthening the Reporting of Observational Studies in Epidemiology) statement for observational studies. Briefly, pregnant women in Japan were recruited for the JECS between January 2011 and March 2014. Women who 1) lived in any of the Study Areas selected by the fifteen Regional Centers located in the country at the time of recruitment; 2) had an expected delivery date after August 1, 2011; and 3) were capable of understanding the Japanese language and completing a self-administered questionnaire were included in the study [10, 12].

**Fig. 1:**
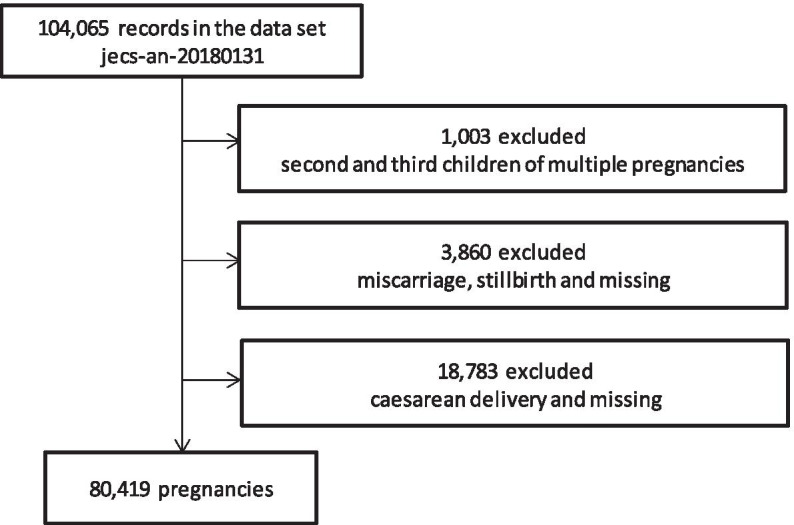
Flow diagram showing the recruitment and exclusion of pregnant women in this study

The present study used the “jecs-ag-20180131” dataset, which was released in March 2018 and contains information on 104,065 fetal records (Fig. 1). Among women with multiple pregnancies during the study period, data for the second or third pregnancy was excluded (n = 1,003); pregnancies with miscarriages, stillbirths or missing data (n = 3,860), and caesarean delivery or missing (n = 18,783) were also excluded. Overall, 80,419 pregnancies were included in the analysis.

The JECS protocol was approved by the Ministry of the Environment’s Institutional Review Board on Epidemiological Studies (no. 100910001) and by the Ethics Committees of all the participating institutions. Written informed consent was obtained from all the study participants.

### Data collection

The study participants completed questionnaires throughout their pregnancies and postpartum periods; i.e., during the first and second/third trimesters, and at one-, six- and twelve-months after delivery. The medical records at the time of registration and just after vaginal delivery or cesarean section were transcribed by doctors, research coordinators, nurses, or midwives.

Information regarding maternal or paternal demographic factors was obtained from the questionnaires completed during pregnancy. Postpartum information was collected from the questionnaires completed during the six months after delivery.

### Outcomes, exposure, and covariates

The primary outcome was the occurrence of postpartum depression. We used the postpartum Edinburgh Postnatal Depression Scores (EPDS) as occurrence of postpartum depressive symptoms at one and six months after delivery and the postpartum K6 scores within one year after delivery as the primary outcomes [13, 14]. The EPDS is a validated, standardized questionnaire consisting of 10 screening items that is commonly used to evaluate for postpartum depressive symptoms. As the cutoff value for the EPDS, we used a score of ≥ 9 as a positive result for postpartum depression [15, 16].

The K6 self-administered questionnaires were assessed using a five-category scale (4 = all the time, 3 = most of the time, 2 = some of the time, 1 = a little of the time, 0 = none of the time), with possible scores ranging from 0–24. According to a Japanese validation study for the K6 questionnaire in the general population, the performance of the K6 questionnaire using an optimal cutoff of ≥ 13 to indicate severe psychological distress was excellent when the performance was examined using an area under the receiver operating characteristic curve (AUC), with values as high as 0.94 (95% confidence interval (CI) = 0.88 to 0.99) [17].

The participants were divided according to mode of delivery into two categories: vaginal delivery with and without anesthesia, including epidural analgesia, spinal-epidural analgesia, or paracervical block.

The covariates included maternal age (categorized as < 20, 20–29, 30–39, ≥ 40 years), maternal body mass index (BMI, categorised as < 18.5, 18.5–25.0, ≥ 25.0 kg/m^2^), maternal educational status (categorised as junior high school or high school, higher professional school or professional school, junior college or college, postgraduate college), annual income (categorised as < 200, 200–400, ≥ 400–600, ≥ 600–800, ≥ 800–1,000 JPY × 10,000; 1 USD = 103.5 JPY, December 2020), recurrent miscarriage (yes vs. no), mode of pregnancy (natural conception vs. others), parity (never vs. ≥ once), drinking history (categorised as never, abstinence before pregnancy, abstinence from this pregnancy, continuance drinking), maternal smoking history (categorised as never, abstinence before pregnancy, abstinence from this pregnancy, continuance smoking 1—10 cigarettes per day, continuance smoking 11—20 cigarettes per day, continuance smoking over 21 cigarettes per day), pre-K6 during first trimester and second/third trimesters (categorised as mentioned above), marriage status at second/third trimester (categorised as married, non-married, divorced, partners’ death) and at six months after delivery (categorised as married, divorced, partners’ death, others), sex of child (categorised as male, female, unclear), Apgar scores at 1 and 5 min (< 7 vs. ≥ 7), inborn errors of metabolism (categorised as nothing, require recheck, require complete check-up, confirm the diagnosis), neonatal anomalies (yes vs. no), breast- or bottle-feeding, frequency of infant crying (categorised as cry well and keep crying, sometimes but stop soon, not too much), and cooperation of partner with nurturing at one month (categorised as always, sometimes, very little, nothing) and at one year after delivery (categorised as nothing, very little, sometimes, well, very well).

### Data analysis

The maternal and postpartum demographic characteristics of the participants were shown with the proportion for discrete data. The Fisher exact test was used to compare the association between the outcome and each variable. Binomial logistic regression analyses were performed by adding all the covariates to calculate the adjusted ORs (aORs) for the association between mode of delivery and postpartum depression. Since missing data can potentially undermine the scientific credibility of causal conclusions, we applied a multiple imputation method to reduce the potential non-response bias created by missing data and to improve the precision of the estimates when calculating the aORs [18, 19]. A total of 20 models, in which all the available variables were used as predictors and outcomes, were created to estimate the aORs. To prevent multiple comparisons possibly yielding false-positive findings, we adopted the Benjamini–Hochberg method and assessed statistical significances by obtaining the *q*-values adjusted for false discovery rate. All the statistical analyses were performed using IBM SPSS Statistics for Windows, version 24.0 (IBM Corp., Japan).

## Results

### Characteristics of prenatal, neonatal, and postpartum statuses and maternal postpartum outcomes

Tables 1 and 2 summarizes the characteristics of the prenatal, neonatal, and postpartum statuses and the maternal postpartum outcomes. Among the 80,419 pregnancies with vaginal deliveries who were included in the analysis, vaginal delivery without anesthesia occurred in 97.1% (n = 78,082) and vaginal delivery with anesthesia in 2.9% (2,337).

**Table 1:**
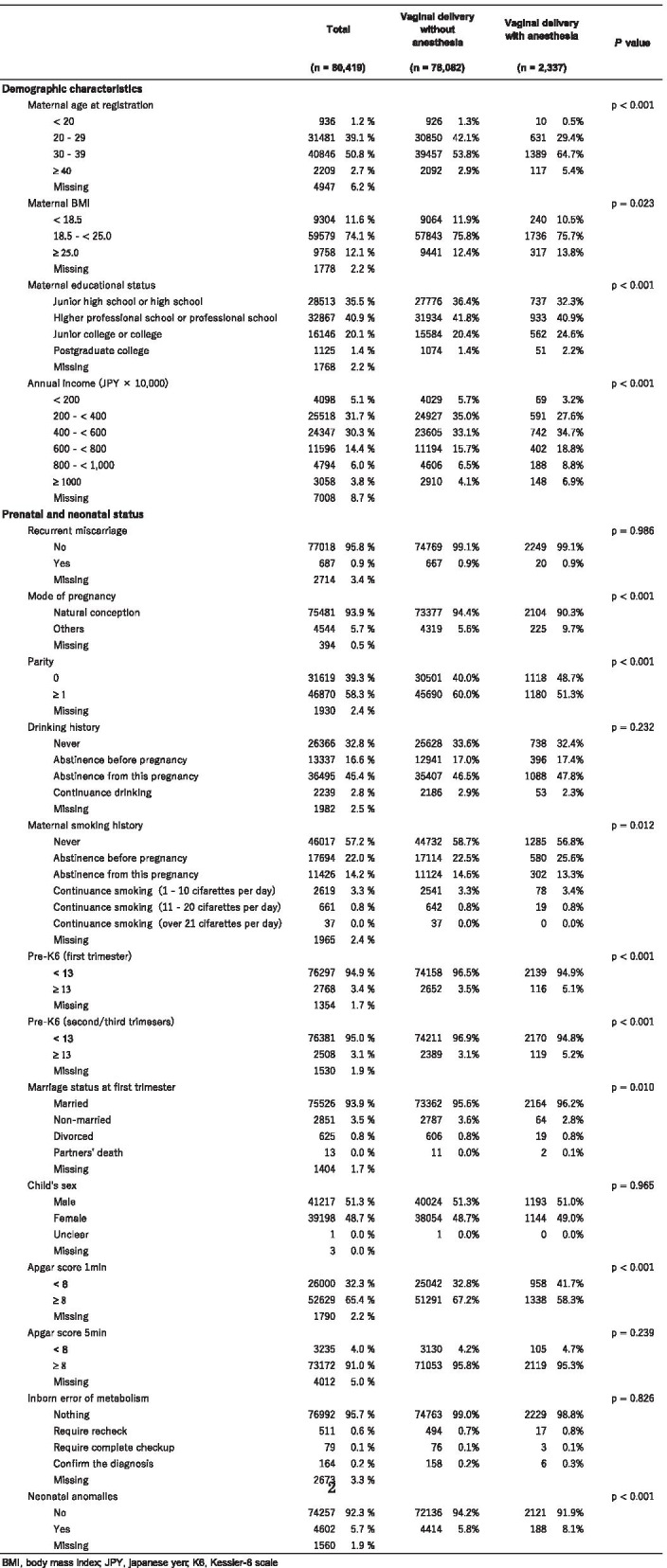
Characteristics of maternal, prenatal and neonatal status

*BMI* Body mass index, *JPY* Japanese yen, *K6* Kessler-6 scale Total Vaginal

**Table 2:**
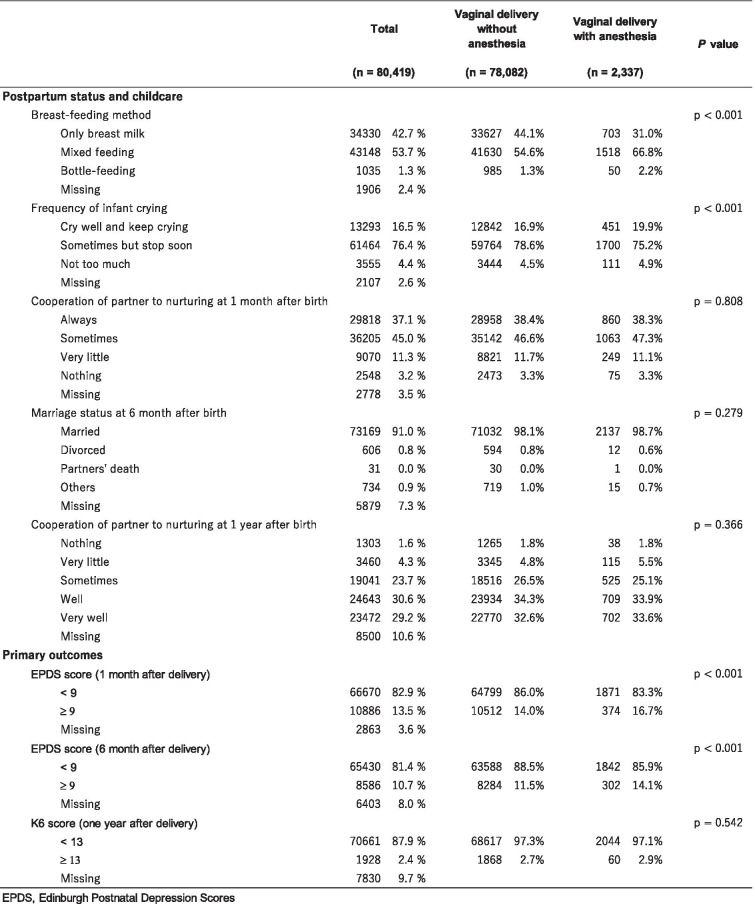
Postpartum status/childcare and primary outcomes

*EPDS* Edinburgh Postnatal Depression Scores Total Vaginal

As for the outcomes, a significant difference in the incidence of postpartum depressive symptoms at one month after delivery was observed according to the mode of delivery (vaginal delivery without analgesia: 14.0%, vaginal delivery with anesthesia: 16.7%, *p* < 0.001). A similar trend was observed at six months after delivery (11.5% and 14.1%, respectively, *p* < 0.001). The K6 scores for postpartum depression at one year after childbirth, however, did not differ significantly according to delivery mode (*p* = 0.542).

With respect to the covariates, the distribution of categorized maternal ages was as follows: 1.2% with an age of < 20 years, 39.1% with an age of 20–29 years, 50.8% with an age of 30–39 years, and 2.7% with an age of ≥ 40 years. All the evaluated demographic characteristics differed significantly according to the vaginal delivery with or without anesthesia (*p* < 0.001). Regarding the prenatal and neonatal statuses, among the women who requested pain control delivery, 5.1% had a positive K6 score for depression during the first trimester, compared with 3.5% in each of the vaginal delivery without analgesia (*p* < 0.001). A similar trend was found during the second/third trimesters, with 5.2% of the women in the delivery with anesthesia, 3.1% of the women in the vaginal delivery without analgesia. As for the postpartum and childcare variables, the breast-feeding method, and frequency of infant crying differed significantly between the two groups (all *p* < 0.001), whereas no significant differences in marriage status at six months after delivery (*p* = 0.279) and partner’s cooperation with nurturing at one month after birth and one year after birth (*p* = 0.808; *p* = 0.366, respectively) were seen.

### Association between delivery with anesthesia and occurrence of postpartum depressive symptoms

The association between delivery with anesthesia and postpartum depression is shown in Table 3. At six months after delivery, women who requested analgesia for delivery with anesthesia had a higher risk of occurrence of postpartum depressive symptoms (aOR: 1.233, 95% CI: 1.079–1.409, *q* = 0.004), compared with vaginal delivery without analgesia. Nevertheless, the association dropped off at one year after delivery. Although no significant difference in the point estimates with or without using the multiple imputation method was seen, the confidence intervals for the aORs calculated using multiple imputation were narrower than those calculated without multiple imputation.

**Table 3:**
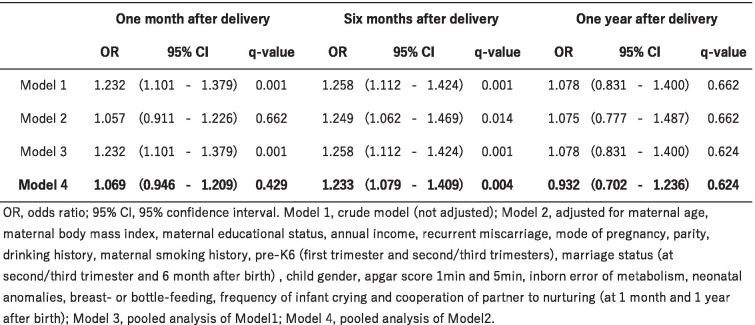
Association of delivery with anesthesia and postpartum depression

*OR* Odds ratio, *95% CI* 95% confidence interval. Model 1, crude model (not adjusted); Model 2, adjusted for maternal age, maternal body mass index, maternal educational status, annual income, recurrent miscarriage, mode of pregnancy, parity, drinking history, maternal smoking history, pre-K6 (first trimester and second/third trimesters), marriage status (atsecond/third trimester and 6 month after birth), child gender, apgar score 1 min and 5 min, inborn error of metabolism, neonatalanomalies, breast- or bottle-feeding, frequency of infant crying and cooperation of partner to nurturing (at 1 month and 1 year after birth); Model 3, pooled analysis of Model1; Model 4, pooled analysis of Model2

## Discussion

The present study found an increased risk of occurrence of postpartum depressive symptoms at six months among women who requested vaginal delivery with anesthesia in Japan. Little consensus exists regarding the effect of delivery with anesthesia on postpartum depression, since the results of previous studies are inconsistent. A recent report suggested that epidural analgesia during labor is not associated with a decreased risk of developing postpartum depression [20]. In contrast, Riazanova et al. reported that postpartum depression was diagnosed at six weeks after delivery in 4.67% of women who requested epidural analgesia, compared with 6.79% among women without analgesia during delivery [21]. Several lines of evidence have suggested that the risk of occurrence of postpartum depressive symptoms is reduced in women who receive epidural analgesia, compared with those without analgesia [22, 23]. Liu et al. reported that the use of neuraxial analgesia during labor was associated with a reduced risk of postpartum depression at two years after delivery [9].

One possible reason for the conflicting reports mentioned above might be due to the nature of the evaluation period for assessing postpartum depression. Postpartum depression is defined as a form of major depression beginning within 4 weeks after delivery and potentially lasting for months or years. In previous studies, the association between postpartum depression and mode of delivery was assessed at time points ranging from a few weeks to as long as two years after delivery. An assessment of the temporal trajectory of postpartum depression using a longitudinal study, rather than cross-sectional assessments at specific time periods, is thus needed.

Another explanation might be the use of different screening tools to evaluate postpartum depression in the previous studies. Both the K6 and the EPDS are commonly used universal screening tools for the occurrence of postpartum depressive symptoms. A systematic review validating the EPDS in postpartum women reported that the sensitivity of the tool ranged widely from 34 to 100%, while the specificity ranged from 44 to 100% [24]. A study comparing the performances of mental health screening tools showed that the EPDS had the highest area under the curve value [25], meaning a high sensitivity for the detection of postpartum depression, while the K6 showed a good balance between sensitivity (74%) and specificity (85%), reaching a sufficient positive predictive value. However, the cutoff values depended on the language of translation, and such differences might be responsible for the discrepant results.

Next, special attention should be paid to the presence of psychological distress before or during early pregnancy and the relations between such factors and the selection of delivery with anesthesia. In Japan, the number of pregnant women who request pain control is relatively small, whereas the rates of delivery with analgesia range between 20 and 70% internationally [7]. Thus, we think that the results of the present study may differ from those of comparable international studies, although not accounting for history of depression should be thought as a weakness of the study. As mentioned in the Introduction, labor with analgesia is uncommon in Japan because of the popular belief that enduring the pain of labor is virtuous. Recently, however, both the number of women of advanced maternal age and the number of pregnant women requesting delivery with anesthesia have been increasing in Japan.

In the present study, the proportion of women with a positive K6 score during their first trimester was higher in the vaginal delivery with anesthesia (5.1%) than in the vaginal delivery without analgesia (3.5%) groups. However, the current study adjusted for the possibility of such an effect on the association between the mode of delivery and postpartum depression using logistic regression analyses. Additionally, as a practical implication, it should be noted that pregnant women who requested delivery with anesthesia had higher K6 scores for depression during the first trimester, compared with women in the delivery without anesthesia.

Depression is the most common psychological disorder in women after childbirth, occurring in 9.0% of pregnant women in Japan (Ministry of Health, Labour and Welfare, 2015 [26]). A national project to prevent postpartum depression has been started in Japan, and postpartum depression is regarded as an essential health issue. In contrast, Olieman et al. reported that women who underwent elective cesarean sections had significantly higher symptom levels of posttraumatic stress disorder and depression than women undergoing vaginal delivery without analgesia [27]. Such discrepancies persist, and health professionals should pay careful attention to all postpartum women, regardless of the use of analgesia.

### Strengths and limitations

The JECS, with 100,000 participants, is the largest nationwide birth cohort study to be conducted in Japan and is considered to be representative of the general population [10, 28]. The outcome measurements were reliable because pregnancy and delivery information were based on medical records transcribed by doctors, research coordinators, nurses, and midwives. Furthermore, the risk estimates for the effect of delivery with anesthesia on postpartum depression were calculated using multiple imputations, providing a high level of scientific credibility and reducing the potential non-response bias created by missing data.

The present study had some limitations. As stated above, two different indexes, the EPDS and the K6 score, were used to evaluate postpartum depression. Since the researchers were unable to implement the use of appropriate indicators for individual studies in their own surveys, the same screening tool could not be used at each measurement point. Although previous studies have shown that the cutoff values for both indicators were appropriate [25], this may have created a potential for systematic bias. It is possible that the women included in this study may have experienced more depressive symptoms before delivery or felt unprepared and, thus, unsuccessful when they requested pain relief.

Given the significantly lower numbers of epidural anesthesia in the study country of origin compared to others (such as the United States), one has to consider whether or not there is a cultural attitude or bias towards this decision. It may be possible women feel ashamed or weak at having made this request. If women feel that they somehow “failed” by making the request for anesthesia, they could be at increased risk for postpartum depression. Overall, the findings in this study may not be generalizable would be generalizable to a country with a higher epidural anesthesia rate, given the potential differences in cultural attitudes/normalization toward this request.

## Conclusion

Delivery with anesthesia was associated with an increased risk for postpartum depressive symptoms at six months after delivery among pregnant women in Japan. Further analysis of maternal environmental statuses and comparing older and younger women who request delivery with anesthesia are needed to determine in which situations might epidural delivery be desirable. Because of prevailing maternal myths, Japan may represent a special environment where deliveries with anesthesia are extremely rare. Unlike in other countries, a higher proportion of women with occurrence of postpartum depressive symptoms at six months after delivery was seen among women requesting delivery with anesthesia; the importance of follow up and screening for postpartum depressive symptoms at 6 months postpartum is thus important for these women.

## Data Availability

Regarding data of the paper publication (http://www.env.go.jp/chemi/ceh/en/index.html). Data availability: Data are unsuitable for public deposition due to ethical restrictions and legal framework of Japan. It is prohibited by the Act on the Protection of Personal Information (Act No. 57 of 30 May 2003, amendment on 9 September 2015) to publicly deposit the data containing personal information. Ethical Guidelines for Medical and Health Research Involving Human Subjects enforced by the Japan Ministry of Education, Culture, Sports, Science and Technology and the Ministry of Health, Labour and Welfare also restricts the open sharing of the epidemiologic data. All inquiries about access to data should be sent to: jecs-en@nies.go.jp. The person responsible for handling enquiries sent to this e-mail address is Dr Shoji F. Nakayama, JECS Programme Office, National Institute for Environmental Studies.

## Abbreviations

EPDS: Edinburgh Postnatal Depression Scores
JECS: The Japan Environment and Children’s Study
K6: Kessler-6 scale
